# The Molecular Mechanism of Potassium Absorption, Transport, and Utilization in Rice

**DOI:** 10.3390/ijms242316682

**Published:** 2023-11-24

**Authors:** Wenli Lian, Anjing Geng, Yihan Wang, Minghao Liu, Yue Zhang, Xu Wang, Guang Chen

**Affiliations:** 1 Institute of Quality Standard and Monitoring Technology for Agro-Products of Guangdong Academy of Agricultural Sciences, Guangzhou 510640, China; 2 Key Laboratory of Testing and Evaluation for Agro-Product Safety and Quality, Ministry of Agriculture and Rural Affairs, Guangzhou 510640, China; 3 Guangdong Provincial Key Laboratory of Quality & Safety Risk Assessment for Agro-Products, Guangzhou 510640, China

**Keywords:** rice, potassium, absorption and transport, molecular mechanism

## Abstract

Potassium is essential for plant growth and development and stress adaptation. The maintenance of potassium homeostasis involves a series of potassium channels and transporters, which promote the movement of potassium ions (K^+^) across cell membranes and exhibit complex expression patterns and regulatory mechanisms. Rice is a major food crop in China. The low utilization rate of potassium fertilizer limits the yield and quality of rice. Elucidating the molecular mechanisms of potassium absorption, transport, and utilization is critical in improving potassium utilization efficiency in rice. Although some K^+^ transporter genes have been identified from rice, research on the regulatory network is still in its infancy. Therefore, this review summarizes the relevant information on K^+^ channels and transporters in rice, covering the absorption of K^+^ in the roots, transport to the shoots, the regulation pathways, the relationship between K^+^ and the salt tolerance of rice, and the synergistic regulation of potassium, nitrogen, and phosphorus signals. The related research on rice potassium nutrition has been comprehensively reviewed, the existing research foundation and the bottleneck problems to be solved in this field have been clarified, and the follow-up key research directions have been pointed out to provide a theoretical framework for the cultivation of potassium-efficient rice.

## 1. Introduction

Potassium (K) is the most abundant cation in plant cells, accounting for 2–10% of plant dry weight [1,2]. Potassium is essential for plant growth and development as it regulates enzyme activity, photosynthetic efficiency, and osmotic stress response [3]. Furthermore, potassium promotes the development of plant sink organs, and the supply of carbon assimilates in sink tissues, thereby increasing crop yield [4,5]. Potassium also plays a role in plant root growth and development, stomatal movement, and reactive oxygen species (ROS) metabolism [6]. Studies have demonstrated that sufficient potassium promotes plant resistance to diseases (including rice blast) [7]. Potassium ions (K^+^) have high fluidity in plants, allowing them to travel vast distances through the xylem and phloem and swiftly transition from old to new leaf tissues. These processes are primarily driven through K^+^ channels or potassium transporters on the plasma membrane [8,9]. Potassium deficiency on cultivated land in China results in a low potassium utilization rate, limiting the sustainable development of agricultural production [10]. Therefore, the urgent need to improve K^+^ utilization efficiency in plants is pegged on in-depth research of the molecular mechanism of plant response to K^+^ deficiency. Plants respond to variations in external K^+^ concentration. As a signal molecule, K^+^ is delivered to the cytoplasm via K^+^ channels or potassium transporters, and the complex regulatory network maintains K^+^ homeostasis in cells, boosting the adaptability to potassium deficiency. As a result, elucidating the molecular regulatory mechanisms of K^+^ channels and potassium transporters has traditionally been the research focus in this field.

Five families (shaker-type K^+^ channel, TPK-type K^+^ channel, HKT transporter, CPA reverse transporter, and KUP/HAK/KT transporter) have been revealed to be involved in K^+^ transport in plants, with shaker-type K^+^ channels and KUP/HAK/KT transporters being particularly significant [11,12,13]. Current research on K^+^ channels and potassium transporters primarily focuses on the model plant *Arabidopsis thaliana*, with limited reports in rice. The present review systematically describes and summarizes the biological functions and regulatory networks of K^+^ channels and transporters in rice and provides a theoretical basis for cultivating novel potassium-efficient rice cultivars.

## 2. Expression Patterns of K^+^ Channels and Transporters in Rice

The structure of shaker-type K^+^ channel proteins in plants and Drosophila is highly similar [14]. Plant shaker-type K^+^ channels are classified into inward K^+^, outward K^+^, and weak rectifier K^+^ channels based on voltage dependency and K^+^ transmembrane movement direction [15]. The *Arabidopsis* genome contains nine shaker-type K^+^ channels, of which *AtAKT1* is the earliest cloned, mainly expressed in root hairs and the root endodermis [16,17]. *AtAKT2* is mainly expressed in the phloem and xylem of the shoot [18]. *AtSKOR* and *AtGORK* are mainly expressed in vascular tissues of *Arabidopsis* roots [19]. *AtKAT1*, *AtKAT2* and *AtKAT3* are mainly expressed in guard cells [20]. The rice genome contains seven shaker-type K^+^ channels, with them being most expressed in the shoots [21,22,23] (Table 1). The inward K^+^ channel *OsAKT1* is primarily expressed in the roots, mediating K^+^ absorption [21,22]. *OsAKT2* is mainly expressed in mature leaves, sheaths, internodes, and glumes [23]. The outward K^+^ channel *OsSKOR* is primarily expressed in the vascular tissues of rice roots, flowers, and seed shields, whereas *OsGORK* is expressed in various rice tissues but is most abundant in flowers [24,25]. *OsKAT1* expression is nearly undetectable in rice roots and stems, but the expression levels of *OsKAT2* and *OsKAT3* are higher in leaves and sheaths [26]. Although the expression patterns of shaker-type K^+^ channels in *Arabidopsis* and rice are similar, their functions in participating in biotic and abiotic stress responses are different [7,22,27].

Six TPK family genes were found in *Arabidopsis thaliana*. Among them, *AtTPK1* was highly expressed in the root tip, vascular tissue, and pollen, *AtTPK3* was mainly expressed in the root tip and pollen, *AtTPK4* was highly expressed in pollen, and *AtTPK5* and *AtKCO3* were expressed in the vascular tissue [28]. Rice contains two members of the TPK family, *OsTPKa* and *OsTPKb*, which are expressed in nearly all tissues [29] (Table 1).

There are 13 HAK/KUP/KT family members in *Arabidopsis thaliana*. *AtKUP2* is highly expressed in flowers. *AtKUP3* and *AtKUP4* are highly expressed in developing siliques. *AtKUP5*, *AtKUP6*, *AtKUP7*, *AtKUP8*, *AtKUP10,* and *AtKUP12* are expressed in roots and leaves. *AtKUP11* has a higher expression level in the reproductive growth stage [30]. *AtHAK5* is the most widely studied in this family, and its expression level in roots is higher [31,32,33]. HAK/KUP/KT is the largest K^+^ transporter family in rice, with 27 members [34] (Table 1). Rice HAK family members, including *OsHAK1*, *OsHAK5*, *OsHAK7*, *OsHAK8*, *OsHAK12*, *OsHAK16*, and *OsHAK18,* have higher expression levels in roots [34,35,36,37,38,39,40,41,42,43,44] (Table 1). *OsHAK5* is expressed in numerous rice tissues, with higher expression levels in the epidermis, vascular tissue, and mesophyll cells of the root system [38]. *OsHAK21* is highly expressed in the roots and leaves of seedlings, as well as in the vascular bundles of anthers and leaf sheaths [45]. *OsHAK26* is primarily expressed in rice anthers and seed coats [46]. The response of *AtHAK5* and *OsHAK5* to low potassium stress was similar [32,33], and both were induced by high salt [34,39], indicating that both genes may be involved in the maintenance of low potassium homeostasis and salt stress response.

Plant HKT transporters mediate K^+^ transport, Na^+^ transport, and Na^+^-K^+^ co-transport. There is only one specific Na^+^ transporter gene *AtHKT1;1* in *Arabidopsis*, which is mainly expressed in the vascular system of the roots and leaves [47]. The rice genome contains eight *OsHKT* genes (Table 1). *OsHKT1;1* is primarily expressed in the leaf phloem [48]. *OsHKT1;3* is highly expressed in the rice shoot [49]. *OsHKT1;4* is primarily expressed in leaf sheaths [50]. *OsHKT1;5* is highly expressed in rice roots [51]. *OsHKT2;1* is expressed in the cortex, endodermis, and the vascular bundle sheath of roots [52]. *OsHKT2;4* is expressed in all rice tissues [53] (Table 1). The HKT1 family genes in rice are expressed in roots and shoots, which is similar to the expression pattern of *AtHKT1;1* in *Arabidopsis*. However, the HKT2 family gene *OsHKT2;1* in rice is induced by low potassium and participates in the absorption of Na^+^ in roots [52], indicating that HKT family genes are involved in the regulation of Na^+^/K^+^ homeostasis in plants.

The NHX transporters are Na^+^/H^+^ antiporters located on the vacuolar membrane. They have been studied in *Arabidopsis thaliana* in depth and have eight members. Among them, *AtNHX1* and *AtNHX2* have high homology and are mainly expressed in roots, shoots, and seedlings. The expression levels of *AtNHX3*, *AtNHX4,* and *AtNHX6* in roots and shoots are low [54]. There are five members in rice; *OsNHX1* and *OsNHX2* exhibit higher transcription levels in rice panicles and flag leaf sheaths. *OsNHX5* is significantly expressed in flag leaves [55] (Table 1). The expression of NHX family genes in *Arabidopsis* and rice was significantly induced by high salt, indicating that they were involved in the salt stress response of plants. The specific functions will be elaborated upon in the subsequent chapters. 

The temporal and spatial expression patterns of the above K^+^ channel and transporter family members and their responses to external potassium concentrations were significantly different, indicating that they have unique and diverse functions in the maintenance of potassium homeostasis and plant growth and development in rice. The biological functions and regulatory mechanisms of related genes will be described in detail from the aspects of root K^+^ absorption, root K^+^ transport, K^+^ transport to the shoot, and K^+^ distribution in various organs.

## 3. K^+^ Absorption and Transport in Rice Roots

### 3.1. K^+^ Absorption in Rice Roots

Plant roots have the most direct touch with the soil and are responsible for the majority of nutrient absorption. As a result, the roots first perceive changes in the external K^+^ concentration. The absorption of water and other nutrients from the soil by *Arabidopsis* occurs first through the epidermal, cortical, and endodermal cells of the root system and then into the vascular tissue [56]. Low potassium can promote the formation of Casparian strips in the endodermis of the roots [57,58,59]. As an important physical barrier, the Casparian strip controls the entry of water and nutrients into the stele through the endodermis cells [60]. The expression of *AtCIPK25* was significantly induced in the root endodermis under anaerobic conditions. AtCIPK25 regulates potassium homeostasis and enhances stress resistance under hypoxic conditions by interacting with AtAKT1 [61].

In addition to the direct effect of root architecture on K^+^ uptake by plants, the K^+^ absorption system also plays an important role. Plants have two distinct K^+^ absorption systems: the low-affinity potassium system is primarily involved when the K^+^ concentration exceeds 1.0 mM, whereas the high-affinity potassium system functions when the external K^+^ concentration is less than 0.2 mM [62,63]. In general, the high-affinity potassium transport system belongs to the active absorption process of reverse chemical gradient and energy consumption, which is primarily mediated by potassium transporters, whereas the low-affinity potassium transport system belongs to the passive absorption process, which is primarily achieved by potassium channels [64]. Mounting research has established that plants respond to variations in external K^+^ concentrations via these two differing affinity potassium absorption systems [65]. 

**Figure 1:**
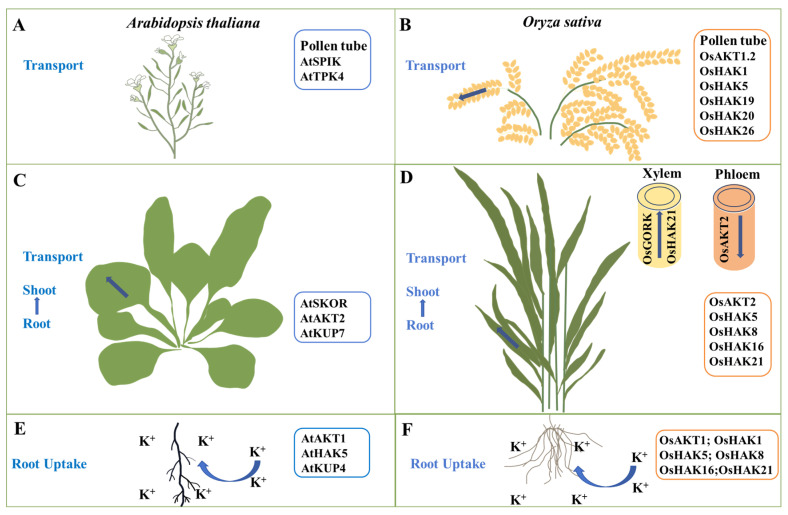
K^+^ channels and transporters involved in K^+^ uptake and transport in *Arabidopsis* and rice. (**A**,**B**) The K^+^ channels or transporters that mediate K^+^ transport in pollen tubes in *Arabidopsis* (**A**) or rice (**B**). (**C**,**D**) The K^+^ channels or transporters involved in K^+^ transport from the roots to shoots in *Arabidopsis* (**C**) or rice (**D**). (**E**,**F**) The K^+^ channels or transporters involved in root K^+^ uptake in *Arabidopsis* (**E**) or rice (**F**).

Shaker-type K^+^ channels and KUP/HAK/KT transporters in higher plants are critical for root K^+^ uptake (Figure 1). AtAKT1 is the first cloned shaker-type K^+^ channel in *Arabidopsis*, and it mediates K^+^ uptake by the roots [66]. OsAKT1 is the rice homolog of AtAKT1 and belongs to the inward K^+^ channel located on the plasma membrane, and being primarily expressed in the roots, mediates K^+^ absorption in rice roots [21]. The decrease in K^+^ absorption and content in the *osakt1* rice mutant leads to the sensitivity of the mutant to low potassium and the inhibition of growth and development [21]. Overexpression of *OsAKT1* increases rice tolerance to osmotic and drought stress by boosting K^+^ absorption, increasing K^+^ accumulation in the roots, and decreasing the Na^+^/K^+^ ratio [22].

K^+^ uptake in plant roots is facilitated by particular KUP/HAK/KT transporters. The KUP/HAK/KT family genes in *Arabidopsis* affect the growth of root hairs, and AtKUP4 is involved in the absorption of K^+^ by the roots [30]. The high-affinity K^+^ transporter *OsHAK1* is primarily expressed in the epidermis and vascular cells of the root system. Potassium deficiency stimulates K^+^ absorption in the root system and significantly induces *OsHAK1* expression [37]. *OsHAK5* is highly expressed in the root epidermis and stele. OsHAK5 contributes to root K^+^ uptake under low-potassium conditions, and *OsHAK5* overexpression significantly increases K^+^ uptake and transport [38]. OsHAK21 promotes K^+^ absorption and salt tolerance in germinated rice seeds under salt stress [45]. In addition, *OsHAK8* and *OsHAK16* are highly expressed in rice roots and are critical in the absorption and transport of K^+^ [41,43]. AtAKT1 and AtHAK5 play a dominant role in root K^+^ uptake in *Arabidopsis*. AtHAK5 is the only transporter that mediates K^+^ uptake in the roots at very low external K concentrations (<20 μM) [32,33]. However, under low-potassium stress, OsHAK1, OsHAK5, OsHAK8, OsHAK16, and OsHAK21 in rice are involved in high-affinity K^+^ uptake; thus, the following questions arise: which transporter plays a leading role? How do these transporters synergistically regulate K^+^ uptake in rice roots? Further research is still needed.

Similar to other higher plants, rice also has two different types of potassium absorption systems. The inward shaker-type K^+^ channels mainly mediate low-affinity potassium absorption, while the KUP/HAK/KT family is mainly involved in high-affinity potassium absorption in the roots, but this distinction is not absolute. For example, OsHAK1 is also involved in root potassium absorption under 1 mM K^+^ conditions. Future research can focus on how rice roots perceive changes in external K^+^ concentration and accurately regulate the division and collaboration of K^+^ channels and transporters through signal transduction. The optimization of core functional genes or the exploration of excellent allelic variations can maximize the range of external K^+^ concentration that can be absorbed by the roots and improve the utilization efficiency of soil potassium.

### 3.2. K^+^ Transport from Rice Roots to Shoots

K^+^ absorbed by plant roots is transferred to the shoots via xylem K^+^ channels and K^+^ transporters [67]. The outward K^+^ channel AtSKOR in *Arabidopsis* mediates K^+^ transport from the xylem to the shoots [68]. Nitrate transporter AtNRT1.5 may indirectly affect K^+^ transport from the roots to shoots through AtSKOR [69]. The *Arabidopsis* K^+^ transporter AtKUP7 is also involved in the transport of K^+^ in the xylem, affecting the long-distance transport of K^+^ [70]. OsGORK plays a crucial role in stomatal movement and K^+^ loading in the xylem in rice. The K^+^ transported from roots to shoots through the xylem is decreased in the *osgork* mutant, as is the K^+^ level in shoots (25% decrease) [25]. Some KUP/HAK/KT transporters also participate in K^+^ transport to the shoots (Figure 1). OsHAK5, OsHAK8, and OsHAK16 promote K^+^ transport from the roots to shoots under K-deficient conditions [38,41,43]. Furthermore, *OsHAK21* is highly expressed in xylem parenchyma and endodermis cells, indicating that OsHAK21 potentially plays a role in K^+^ delivery to the shoots [45]. Low potassium promotes the expression of low-affinity nitrate transporter *OsNPF2.4* in the leaves, in addition to K^+^ channels and K^+^ transporters. The K^+^ content of the *osnpf2.4* mutant increases in a high ${NO}_{3}^{-}$ environment, which indirectly influences K^+^ reuse in the roots and stems, indicating that OsNPF2.4 may mediate the transport and utilization of K^+^ to the shoots [71]. AtSKOR and AtKUP7 in *Arabidopsis* are involved in the long-distance transport of K^+^, while the specific function of OsSKOR in rice has not been reported in detail and needs to be further identified.

To date, only a few K^+^ channels and transporters have been identified in rice that have the function of transporting K^+^ to the shoots. The long-distance transport of K^+^ is the premise of K accumulation in the shoots of rice. It is the basis for the normal growth of plants and improvement of low potassium tolerance. Therefore, in the future, new functional genes involved in K^+^ transport to the shoots of rice can be further explored, which is conducive to further improving the molecular mechanism of rice response to low-potassium stress.

## 4. K^+^ Transport in Rice Shoots

### 4.1. K^+^ Transport in Rice Leaves

K^+^ is transported to different tissues and organs following absorption by plant roots for effective utilization via the in vivo transport system. The shaker-type K^+^ channel AtAKT2 in *Arabidopsis* is a weak rectifier K^+^ channel that primarily mediates K^+^ loading or unloading in the phloem, allowing for long-distance transport in plants [72,73]. OsAKT2 is the rice homolog of AtAKT2 and belongs to the weak rectifier K^+^ channel family. OsAKT2 disruption decreases the K^+^ content in phloem sap, indicating that OsAKT2 plays a crucial role in the phloem loading process of K^+^ [23] (Figure 1). The *osakt2* mutant had higher K^+^ accumulation in the old leaves than the wild type (WT) but decreased K^+^ accumulation in the young leaves, indicating that OsAKT2 mediates K^+^ redistribution from the old to the young leaves [23]. The *AKT2* gene in *Arabidopsis* and rice has similar functions and plays a role in the phloem loading of K^+^. OsAKT2 in rice is also involved in the redistribution of K^+^ and improves the utilization efficiency of K^+^, which is beneficial to improving rice yield and quality.

### 4.2. K^+^ Transport in Rice Guard Cells

The stoma is a critical location in plant leaves for water vapor exchange and CO_2_ acquisition. The number, density, and opening and closing of stomata are crucial indicators of plant drought stress response, and K^+^ channels and K^+^ transporters in rice guard cells can regulate stomatal opening [74]. Guard cells in *Arabidopsis thaliana* have elevated expression levels of the inward shaker-type K^+^ channels *AtKAT1* and *AtKAT2* and the outward K^+^ channel *AtGORK*; these channels regulate stomatal opening and closing by mediating K^+^ transmembrane influx and efflux [75,76]. The shaker-type K^+^ channels, *OsKAT2,* and *OsKAT3* in rice are primarily expressed in guard cells, and the OsKAT2 influx-type K^+^ channel activity is comparable to that of *Arabidopsis* AtKAT1 [26]. There is no typical K^+^ channel activity despite the strong homology between OsKAT3 and OsKAT2. The possibility of restoring the channel activity of OsKAT3 by removing its C-terminal domain indicates that the C-terminal region functions as a regulatory domain suppressing channel activity [26]. *OsGORK* is expressed in both the guard and subsidiary guard cells of rice stomata and encodes a slowly activated and outwardly rectifying K^+^ channel. The loss of *OsGORK* function decreases K^+^ efflux from guard cells, slows stomatal closure, and increases the rate of water loss [25].

### 4.3. K^+^ Transport during Rice Pollen Development

Pollen development, a crucial aspect of plant sexual reproduction, also significantly affects yield [77]. K^+^ stimulates cell expansion, hydrates tissues, creates swelling pressure, and accelerates the formation of pollen tubes [78,79]. The inward K^+^ channel *AtSPIK* is expressed explicitly in *Arabidopsis* pollen, inhibiting the pollen germination and pollen tube growth of *atspik* mutants [80,81]. The plasma membrane contains the shaker-type K^+^ channel ZmKZM1 in maize and OsAKT1.2 in rice, which regulates pollen development, pollen tube growth, and rupture [82,83] (Figure 1). *Arabidopsis* AtTPK4 regulates K^+^ homeostasis and pollen tube growth [84]. While the functions of OsTPKa and OsTPKb in rice in response to salt stress have been documented, their potential regulatory role in pollen development is unclear [85]. OsHAK1 is involved in K^+^ distribution in rice floral organs. The loss of OsHAK1 function leads to decreased pollen viability and fertility [37]. OsHAK5 is involved in acquiring K^+^ by pollen grains in rice [46]. OsHAK26, found in the Golgi apparatus, is involved in the formation of pollen walls and is critical for pollen development and fertility [46]. The interaction of rice receptor-like kinase OsRUPO (ruptured pollen tube) with OsHAK1, OsHAK19, and OsHAK20 mediates the growth and integrity of pollen tubes by maintaining K^+^ homeostasis [86]. 

The mobility of K^+^ is conducive to its transfer in various organs of rice. K^+^ channels and transporters are involved in the redistribution of K^+^ in rice. At present, most studies focus on revealing the function of K^+^ transporters in an independent organ (such as guard cells and pollen), while there are few studies on the movement or redistribution of K^+^ between multiple tissues or organs. The movement of K^+^ from senescent tissue to newborn tissue can meet the basic growth needs of plants and promote the improvement of low potassium tolerance and potassium utilization efficiency. However, there is a lack of analysis of related functional genes and regulatory networks. Follow-up studies can further explore this and provide feasible solutions for potassium-efficient rice breeding.

## 5. Regulation of K^+^ Channels and Transporters

**Figure 2:**
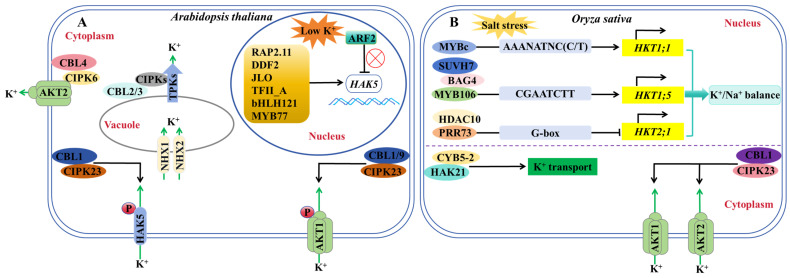
A schematic diagram of the regulatory pathways of K^+^ channels or transporters in *Arabidopsis* and rice. (**A**,**B**) Transcriptional regulation and post-translational regulation pathways of K^+^ channels or transporters in *Arabidopsis* (**A**) or rice (**B**). “→” indicates promoting effects. “⟂” indicates an inhibitory effect.

*Arabidopsis* primarily regulates the expression or activity of downstream functional genes or proteins at the transcriptional and post-transcriptional levels in response to low potassium stress, hence regulating the absorption, transport, and redistribution of K^+^ in plants. The CBL–CIPK complex plays a role in the post-transcriptional regulation of K^+^ channels: AtCBL1/9 interacts with AtCIPK23 and recruits it to the plasma membrane. AtCIPK23 phosphorylates AtAKT1 on the plasma membrane, and this phosphorylation increases in potassium-deficient plants, allowing them to absorb more K^+^ from the environment [87,88] (Figure 2A). The AtCBL4–AtCIPK6 complex plays a role in AtAKT2 regulation, recruiting it from the cytoplasm to the plasma membrane and increasing the AtAKT2-mediated potassium current [73]. The AtCBL2/3-AtCIPK3/9/23/26 complex promotes K^+^ efflux from vacuoles to the cytoplasm by activating the potassium channel TPK on vacuoles [89] (Figure 2A). Although post-transcriptional regulation of KT/KUP/HAK transporters has been documented, including AtHAK5 activation of AtCBL1-AtCIPK23 phosphorylation [90], further research has established that potassium transporters are primarily regulated at the transcriptional level; multiple transcription factors, including AtRAP2.11, AtDDF2, AtJLO, AtTFII_A, AtbHLH121, AtARF2 and AtMYB77, regulate *AtHAK5* expression [91,92] (Figure 2A). The low-potassium phosphorylation of AtARF2 can alleviate its transcriptional inhibition of *AtHAK5*, while AtMYB77 can increase *AtHAK5* expression by binding to the *AtHAK5* promoter, increasing plant high-affinity potassium absorption and low potassium tolerance [93,94]. AtMYB59 is a positive transcription regulator of AtNRT1.5. The transcription and protein levels of AtMYB59 are reduced, and *AtNRT1.5* expression is suppressed under low-potassium stress, resulting in a blockage of K^+^ transport from *Arabidopsis* roots to the crown [95]. In addition, yeast one-hybrid experiments in wheat and maize revealed that TaNAC71 interacted with the *TaHAK1* promoter, while ZmRAP2.11 and ZmARF2 interacted with the *ZmHAK1* promoter [96,97].

Until recently, the molecular regulation pathway of potassium nutrition in rice has been mainly reported at the post-transcriptional level: OsCBL1-OsCIPK23 regulates the activity of OsAKT1 and OsAKT2 [21,98]. OsHAK21 interacts with the OsCYB5-2 protein to stabilize OsHAK21-mediated K^+^ uptake and to maintain intracellular K^+^/Na^+^ balance [99] (Figure 2B). Transcriptional regulation is solely reported in the potassium transporter HKT. HKT proteins are classified into two categories based on the amino acid differences and transport characteristics in their first pore domain. Class I members (HKT1) are Na^+^ transporters, while class II members (HKT2) are Na^+^/K^+^ co-transporters [100]. Previous research has focused on the transcriptional regulation of *OsHKT1*: OsSUVH7, OsBAG4, and OsMYB106 to form a transcriptional complex to activate *OsHKT1;5* expression under salt stress [101]. OsMYBc binds to the *OsHKT1;1* promoter and regulates Na^+^ accumulation in the shoots [48,102] (Figure 2B).

In rice, only two post-translational regulation pathways of potassium nutrition, OsCBL1-OsCIPK23-OsAKT1/OsAKT2 and OsHAK21-OsCYB5-2, have been found, and only the transcriptional regulation mechanisms of Na^+^ transporter genes *OsHKT1;1*, *OsHKT1;5* and *OsHKT2;1* have been clarified. The fine regulation of potassium nutrition at the transcriptional level in rice and its relationship with low-potassium stress tolerance have not been reported. Therefore, it is of great significance to further explore the key components of the rice potassium nutrition regulation pathway at the transcriptional level and analyze its molecular mechanism to improve the regulatory network of rice in response to low potassium.

## 6. Relationship between Potassium and Salt Stress Response in Rice

Intracellular K^+^/Na^+^ equilibrium is a crucial indicator of salt tolerance when plants are subjected to salt stress [103]. High Na^+^ concentration competes with K^+^ for cell entry, disrupting K^+^/Na^+^ equilibrium. In this view, maintaining a low Na^+^ concentration and normal K^+^/Na^+^ balance is critical for salt stress resistance [104,105,106,107]. Increased K^+^ uptake in high-salt environments can enhance plant salt tolerance [108,109]. In addition, salt stress can also induce an increase in endogenous NO in plants, promote the ion exchange of Na^+^/H^+^, and the accumulation of K^+^ in roots and leaves, thereby improving the salt tolerance of plants [110].

**Figure 3:**
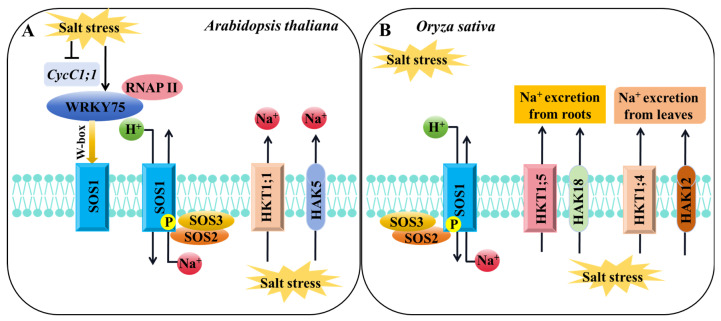
A schematic diagram of the regulatory pathways of K^+^ channels or transporters involved in salt stress response in *Arabidopsis* and rice. (**A**,**B**) The regulatory pathway of K^+^ channels or transporters involved in salt stress response in *Arabidopsis* (**A**) or rice (**B**). “→” indicates promoting effects. “⟂” indicates an inhibitory effect.

HKT transporters are crucial in plant K^+^/Na^+^ transport and salt stress response [111]. Under salt stress, the *athkt1;1 Arabidopsis* mutant accumulated excessive Na^+^ and leaf chlorosis [112]. AtHKT1;1 protected the leaves from salt stress damage by mediating the removal of Na^+^ from the xylem [113]. Rice OsHKT1;1 plays a role in regulating Na^+^ content and reducing Na^+^ toxicity in leaves. The *oshkt1;1* mutant is hypersensitive to salt stress, accumulates more Na^+^ in vivo, and inhibits plant growth [48]. OsHKT1;4 is responsible for Na^+^ unloading in the xylem, mediates Na^+^ transport in the shoots, promotes Na^+^ excretion from the leaves, and improves the salt tolerance of rice during reproductive development [50] (Figure 3). *OsHKT1;5* is primarily expressed in parenchyma cells around the xylem of rice. OsHKT1;5 reflows excessive Na^+^ from the shoots to the roots through xylem unloading under salt stress, lowering Na^+^ toxicity and improving salt tolerance in rice [51]. OsHKT2;1 promotes root Na^+^ absorption under K^+^-deficient conditions, and Na^+^ temporarily substitutes K^+^ to support plant growth [49,52]. The OsPRR73 protein can bind to the promoter of *OsHKT2;1*, inhibit *OsHKT2;1* transcription by recruiting histone deacetylase HDAC10, decrease Na^+^ absorption in a specific time, and regulate the salt tolerance of rice by regulating Na^+^ homeostasis and ROS levels [114]. *AtHKT1;1* in *Arabidopsis* and *OsHKT1;1*, *OsHKT1;4,* and *OsHKT1;5* in rice belong to the class I HKT transporter family, which have high similarity in preventing excessive accumulation of Na^+^ in the leaves and enhancing plant salt tolerance by increasing the K^+^/Na^+^ ratio [51,115] (Figure 3).

HAK transporters also exert crucial functions in plant response to salt stress (Figure 3). Overexpression of *OsHAK1* and *OsHAK5* enhances rice tolerance to salt stress by promoting K^+^ uptake and transport and maintaining K^+^/Na^+^ equilibrium [36,39]. OsHAK12 mediates Na^+^ transport from the roots to shoots to improve salt tolerance under salt stress [42], while OsHAK18 enhances salt tolerance by mediating Na^+^ redistribution from the shoots to roots [44]. OsHAK21 promotes the absorption of K^+^ and Na^+^ during rice seed germination, induces the expression of abscisic acid (ABA) signaling pathway genes, increases ABA biosynthesis, and inhibits ROS accumulation, consequently enhancing salt tolerance during seed germination [45]. *OsHAK20* and *OsHAK13* were identified as critical quantitative trait loci (QTLs) related to salt tolerance during the seedling and flowering stages, respectively, using high-resolution genetic maps. These two genes can be utilized as molecular markers in marker-assisted selection to develop highly resistant rice [116].

In addition, other K^+^ channels and transporters are involved in the regulation of Na^+^/K^+^ homeostasis under salt stress (Figure 3): *OsKAT1* overexpression improves rice salt tolerance by increasing intracellular K^+^ content [117]. The vacuolar Na^+^/H^+^ antiporter NHX family genes play an important role in plant salt stress response. Under high-salt conditions, *AtNHX1* overexpression plants have a stronger ability to maintain intracellular K^+^ homeostasis, thereby improving salt tolerance [118]. Under salt stress, K^+^ accumulation decreased and Na^+^ content increased in the leaves of *nhx1 nhx2* mutants, indicating that AtNHX1 and AtNHX2 may be involved in the maintenance of Na^+^/K^+^ homeostasis [119]. In addition, the *nhx5 nhx6* mutant was also sensitive to salt stress [120]. AtNHX7/AtSOS1 located on the plasma membrane is regulated by the phosphorylation of the upstream AtSOS2–AtSOS3 complex [121] and actively transports Na^+^ to expel Na^+^ from the roots [122]. Under high-salt conditions, the expression of *AtCycC1;1* was inhibited, while the expression of *AtWRKY75* was activated, resulting in increased recruitment of the *AtSOS1* promoter by RNAP II, which promoted the expression of *AtSOS1* and enhanced salt tolerance [123] (Figure 3A). *OsNHX1* and *OsNHX2* are induced by salt stress in rice, and the accumulation of Na^+^ and K^+^ in the cytoplasm is regulated by vacuolar compartmentation. Overexpression of *OsNHX1* significantly improves the salt tolerance of rice [109,111]. In rice, OsSOS3 also interacts with OsSOS2 and phosphorylates the OsSOS2 protein, thereby activating OsSOS1 on the plasma membrane, resulting in Na^+^ efflux from cells and maintaining the Na^+^/K^+^ balance in cells [124] (Figure 3B).

K^+^ channels and transporters in *Arabidopsis* and rice maintain intracellular K^+^/Na^+^ homeostasis and normal turgor pressure by regulating the K^+^/Na^+^ ratio in vivo, prevent cells from being subjected to ion toxicity and oxidative damage, and coordinate the dynamic balance between growth and development and salt stress adaptation.

## 7. Synergistic Regulation of Potassium with Nitrogen and Phosphorus

### 7.1. Synergistic Regulation of Potassium and Nitrogen

The coordinated application of nitrogen and potassium fertilizers can increase the yield and quality of crops [125]. The nitrate transporter AtNRT1.5/NPF7.3 in *Arabidopsis thaliana* is an H^+^/K^+^ antiporter involved in K^+^ xylem loading. The *atnrt1.5* mutant lacks K^+^ transport to the shoots [69,126]. AtCBL1-AtCIPK23 phosphorylates ammonia transporters AtAMT1;1 and AtAMT1;2 to avoid ${NH}_{4}^{+}$ accumulation under an external high concentration of ${NH}_{4}^{+}$ and low potassium conditions [127]. OsNPF2.4 promotes ${NO}_{3}^{-}$ absorption and long-distance transport. *OsNPF2.4* expression in the roots and shoots is regulated under low-potassium conditions, indirectly influencing K^+^ reuse [71]. OsNRT2.4 and OsAMT1;3 are implicated in the absorption of ${NO}_{3}^{-}$ and ${NH}_{4}^{+}$ in rice, respectively. Low potassium (-K) inhibits their expression, while low nitrogen (-N) or low nitrogen and low potassium (-N-K) induce their expression, indicating that the two genes play different roles in the response to -N and -K [128,129,130]. In addition, -N significantly influences the expression of some HAK transporter genes: -N, -K, and -N-K induce the expression of *OsHAK1*, particularly in the shoots under -N-K conditions [130]. -K promotes the expression of *OsHAK5*, *OsHAK16*, and *OsHAK17*, while -N and -N-K significantly inhibit the expression of the three genes [130]. Some OsHAKs transporter genes respond to both -K and -N, indicating that they may be involved in the synergistic regulation of potassium and nitrogen, but the specific signal transduction and regulatory pathways need to be further revealed.

### 7.2. Synergistic Regulation of Potassium and Phosphorus

There are limited investigations on the synergistic effect of phosphorus and potassium. The expression of particular transcription factors, MAPK and MAPKK, is rapidly induced following variations in the external potassium or phosphorus concentrations in tomatoes [131]. Phosphate transporter OsPHT1;4 is involved in Pi absorption in rice and Pi transport and homeostasis maintenance [132]. Low phosphorus (-P), low potassium (-K), and low phosphorus and low potassium (-P-K) were found to significantly induce *OsPHT1;4* expression in roots [130]. OsPHO1;1 and OsPHO1;3 have the function of phosphorus transport. -P induces the two genes, whereas -K and -P-K inhibit their expression [130]. However, the molecular signaling pathways co-regulated by potassium and phosphorus in rice warrant further investigation.

## 8. Conclusions and Future Perspectives

The absorption of K^+^ in rice roots and the transport of K^+^ in vivo involves a series of K^+^ channels and transporters. These transporters promote K^+^ movement across cell membranes and exhibit varying expression patterns, subcellular localization, transport affinity, and regulatory mechanisms. Therefore, they serve distinct and diversified functions in rice growth and development and stress adaptability. However, with the research progress on the cloning and functional identification of K^+^ absorption- and transport-related genes, the intricacy of potassium nutrition trait regulation has steadily been recognized. Gene networks regulate all processes from the signal reception, transduction, gene interaction, and expression of functional genes to physiological or morphological changes related to K^+^ transport and distribution, ultimately modifying K^+^ absorption or utilization efficiency. Therefore, potassium nutrition-efficient breeding must be examined from the perspective of the whole signal regulation network. Of note, only the post-transcriptional regulation and modification of potassium channels and transporters have been reported in rice, with no evidence of upstream critical genes in the transcriptional regulation of potassium nutrition. In addition, the AtMYB59-AtNRT1.5 transcriptional regulatory pathway in *Arabidopsis* is crucial in responding to K^+^/${NO}_{3}^{-}$ deficiency and regulating the synergistic transport of potassium and nitrogen. Are there analogous critical genes regulating the synergistic utilization of potassium and nitrogen in rice? Therefore, it is imperative in the future to further investigate new potassium nutrition regulatory genes, reveal the genetic mechanism of maintaining potassium homeostasis in rice, analyze the regulatory network of the synergistic and efficient utilization of potassium, nitrogen, and phosphorus nutrition in rice, and uncover the excellent allelic variation of key functional genes in germplasm resources, explore its utilization value, and provide genetic resources and theoretical support for cultivating new rice varieties with efficient utilization of nutrients.

## Figures and Tables

**Table 1:** Expression patterns of K^+^ channel and transporter genes in *Arabidopsis* and rice.

Gene Family	Gene	Gene ID	Expression Tissues	References
Shaker K^+^ channels in *Arabidopsis* and rice	*AtAKT1*	AT2G26650	Root	[16,17]
	*AtAKT2*	AT4G22200	Shoot	[18]
	*AtSKOR*	AT3G02850	Root	[19]
	*AtGORK*	AT5G37500	Root	[19]
	*AtKAT1*	AT5G46240	Shoot	[20]
	*AtKAT2*	AT4G18290	Shoot	[20]
	*AtKAT3*	AT4G32650	Shoot	[20]
	*OsAKT1*	Os01g45990	Root	[21,22]
	*OsAKT2*	Os05g35410	Shoot	[23]
	*OsSKOR*	Os04g36740	Root and flower	[24]
	*OsGORK*	Os06g14030	Root and flower	[24,25]
	*OsKAT1*	Os01g55200	/	[26]
	*OsKAT2*	Os01g11250	Shoot and leaf sheath	[26]
	*OsKAT3*	Os02g14840	Shoot and leaf sheath	[26]
TPK-type K^+^ channels in *Arabidopsis* and rice	*AtTPK1*	AT5G55630	Root and pollen	[28]
	*AtTPK3*	AT4G18160	Root and pollen	[28]
	*AtTPK4*	AT1G02510	Pollen	[28]
	*AtTPK5*	AT4G01840	Vascular tissue	[28]
	*AtKCO3*	AT5G46360	Vascular tissue	[28]
	*OsTPKa*	Os03g54100	Root and shoot	[29]
	*OsTPKb*	Os07g01810	Root and shoot	[29]
KUP/HAK/KT transporters in *Arabidopsis* and rice	*AtKUP2*	AT2G40540	Flower	[30]
	*AtKUP3*	AT3G02050	Silique	[30]
	*AtKUP4*	AT4G23640	Silique	[30]
	*AtKUP5*	AT4G33530	Root and shoot	[30]
	*AtKUP6*	AT1G70300	Root and shoot	[30]
	*AtKUP7*	AT5G09400	Root and shoot	[30]
	*AtKUP8*	AT5G14880	Root and shoot	[30]
	*AtKUP11*	AT2G35060	Shoot	[30]
	*AtKUP12*	At1G60160	Root and shoot	[30]
	*AtHAK5*	AT4G13420	Root	[31,32,33,34]
	*OsHAK1*	Os04g32920	Root	[35,36,37]
	*OsHAK5*	Os01g70490	Root	[38,39]
	*OsHAK7*	Os07g47350	Root and stem	[40]
	*OsHAK8*	Os03g21890	Root	[41]
	*OsHAK12*	Os08g10550	Root	[42]
	*OsHAK16*	Os03g37840	Root	[43]
	*OsHAK18*	Os09g38960	Root	[44]
	*OsHAK21*	Os03g37930	Root and shoot	[45]
	*OsHAK26*	Os08g39950	Anther and seed coat	[46]
HKT transporters in *Arabidopsis* and rice	*AtHKT1;1*	AT4G10310	Root and shoot	[47]
	*OsHKT1;1*	Os04g51820	Shoot	[48]
	*OsHKT1;3*	Os02g07830	Bud	[49]
	*OsHKT1;4*	Os04g51830	Leaf sheath	[50]
	*OsHKT1;5*	Os01g20160	Root	[51]
	*OsHKT2;1*	Os06g48810	Root	[52]
	*OsHKT2;4*	Os06g48800	Root and shoot	[53]
Vacuolar Na^+^/H^+^ antiporters in *Arabidopsis* and rice	*AtNHX1*	AT5G27150	Root and shoot	[54]
	*AtNHX2*	AT3G05030	Root and shoot	[54]
	*AtNHX3*	AT5G55470	Root and shoot	[54]
	*AtNHX4*	AT3G06370	Root and shoot	[54]
	*AtNHX6*	AT1G79610	Root and shoot	[54]
	*OsNHX1*	Os07g47100	Panicle and leaf sheath	[55]
	*OsNHX2*	Os05g05590	Panicle and leaf sheath	[55]
	*OsNHX5*	Os09g11450	Flag leaf	[55]

(“/” indicates not yet reported).

## Data Availability

Not applicable.

## References

[1] Very A.A., Sentenac H. (2003). Molecular mechanisms and regulation of K^+^ transport in higher plants. Annu. Rev. Plant Biol..

[2] Shin R. (2014). Strategies for improveing potassium use efficiency in plants. Mol. Cells.

[3] Clarkson D.T., Hanson J.B. (1980). The mineral nutrition of higher plants. Annu. Rev. Plant Physiol..

[4] Wang M., Zheng Q.S., Shen Q.R., Guo S.W. (2013). The critical role of potassium in plant stress response. Int. J. Mol. Sci..

[5] Pan Y.H., Lu Z.F., Lu J.W., Li X.K., Cong R.H., Ren T. (2017). Effects of low sink demand on leaf photosynthesis under potassium deficiency. Plant Physiol. Biochem..

[6] de Bang T.C., Husted S., Laursen K.H., Persson D.P., Schjoerring J.K. (2021). The molecular-physiological functions of mineral macronutrients and their consequences for deficiency symptoms in plants. New Phytol..

[7] Shi X.T., Long Y., He F., Zhang C.Y., Wang R.Y., Zhang T., Wu W., Hao Z.Y., Wang Y., Wang G.L. (2018). The fungal pathogen *Magnaporthe oryzae* suppresses innate immunity by modulating a host potassium channel. PLoS Pathog..

[8] Gajdanowicz P., Michael E., Sandmann M., Rocha M., Corrêa L.G., Ramírez-Aguilar S.J., Gomez-Porras J.L., González W., Thibaud J.B., Dongen J.T. (2011). Potassium (K^+^) gradients serve as a mobile energy source in plant vascular tissues. Proc. Natl. Acad. Sci. USA.

[9] Wigoda N., Moshelion M., Moran N. (2014). Is the leaf bundle sheath a smart flux value for K^+^ nutrition?. J. Plant Physiol..

[10] Wang Y., Wu W.H. (2015). Genetic approaches for improvement of the crop potassium acquisition and utilization efficiency. Curr. Opin. Plant Biol..

[11] Grabov A. (2007). Plant KT/KUP/HAK potassium transporters: Single family-Multiple functions. Ann. Bot..

[12] Bohm J., Messerer M., Muller H., Scholz-Starke J., Gradogna A., Scherzer S., Maierhofer T., Bazihizina N., Zhang H.M., Stigloher C. (2018). Understanding the molecular basis of salt sequestration in epidermal bladder cells of Chenopodium quinoa. Curr. Biol..

[13] Ragel P., Raddatz N., Leidi E.O., Quintero F.J., Pardo J.M. (2019). Regulation of K^+^ nutrition in plants. Front. Plant Sci..

[14] Papazian D.M., Schwarz T.L., Tempel B.L., Jan L.Y. (1987). Cloning of genomic and complementary DNA from shaker, a putative channel gene from Drosphila. Science.

[15] Wang Y., Wu W.H. (2013). Potassium transport and signaling in higher plants. Annu. Rev. Plant Biol..

[16] Pilot G., Gaymard F., Mouline K., Chérel I., Sentenac H. (2003). Regulated expression of Arabidopsis shaker K^+^ channel genes involved in K^+^ uptake and distribution in the plant. Plant Mol. Biol..

[17] Pilot G., Pratelli R., Gaymard F., Meyer Y., Sentenac H. (2003). Five-group distribution of the Shaker-like K^+^ channel family in higher plants. J. Mol. Evol..

[18] Deeken R., Ivashikina N., Czirjak T., Philippar K., Becker D., Ache P., Hedrich R. (2003). Tumour development in Arabidopsis thaliana involves the Shaker-like K^+^ channels AKT1 and AKT2/3. Plant J..

[19] Gaymard F., Pilot G., Lacombe B., Bouchez D., Bruneau D., Boucherez J., Michaux-Ferrière N., Thibaud J.B., Sentenac H. (1998). Identification and disruption of a plant shaker-like outward channel involved in K^+^ release into the xylem sap. Cell.

[20] Ronzier E., Corratgé-Faillie C., Sanchez F., Prado K., Brière C., Leonhardt N., Thibaud J.B., Xiong T.C. (2014). CPK13, a noncanonical Ca_2_^+^-dependent protein kinase, specifically inhibits KAT2 and KAT1 shaker K^+^ channels and reduces stomatal opening. Plant Physiol..

[21] Li J., Long Y., Qi G.N., Li J., Xu Z.J., Wu W.H., Wang Y. (2014). The OsAKT1 channel is critical for K^+^ uptake in rice roots and is modulated by the rice CBL1-CIPK23 complex. Plant Cell.

[22] Ahmad I., Mian A., Maathuis F.J.M. (2016). Overexpression of the rice AKT1 potassium channel affects potassium nutrition and rice drought tolerance. J. Exp. Bot..

[23] Tian Q.X., Shen L.K., Luan J.X., Zhou Z.Z., Guo D.S., Shen Y., Jing W., Zhang B.L., Zhang Q., Zhang W.H. (2021). Rice shaker potassium channel OsAKT2 positively regulates salt tolerance and grain yield by mediating K^+^ redistribution. Plant Cell Environ..

[24] Kim H.Y., Choi E.H., Min M.K., Hwang H., Moon S.J., Yoon I., Byun M.O., Kim B.G. (2015). Differential gene expression of two outward-rectifying shaker-like potassium channels OsSKOR and OsGORK in rice. J. Plant Biol..

[25] Nguyen T.H., Huang S.G., Meynard D., Chaine C., Michel R., Roelfsema M., Guiderdoni E., Sentenac H., Véry A.A. (2017). A dual role for the OsK5.2 ion channel in stomatal movements and K^+^ loading into xylem sap. Plant Physiol..

[26] Hwang H., Yoon J., Kim H.Y., Min M.K., Kim J.A., Choie E.H., Lan W.Z., Bae Y.M., Luan S., Cho H. (2013). Unique features of two potassium channels, OsKAT2 and OsKAT3, expressed in rice guard cells. PLoS ONE.

[27] Li J.F., Shen L.K., Han X.L., He G.F., Fan W.X., Li Y., Yang S.P., Zhang Z.D., Yang Y.Q., Jin W.W. (2023). Phosphatidic acid-regulated SOS2 controls sodium and potassium homeostasis in Arabidopsis under salt stress. EMBO J..

[28] Voelker C., Schmidt D., Mueller-Roeber B., Czempinski K. (2006). Members of the Arabidopsis AtTPK/KCO family form homomeric vacuolar channels in planta. Plant J..

[29] Isayenkov S., Isner J.C., Maathuis F.J. (2011). Rice two-pore K^+^ channels are expressed in different types of vacuoles. Plant Cell.

[30] Ahn S.J., Shin R., Schachtman D.P. (2004). Expression of KT/KUP genes in Arabidopsis and the role of root hairs in K^+^ uptake. Plant Physiol..

[31] Nieves-Cordones M., Lara A., Ródenas R., Amo J., Rivero R.M., Martínez V., Rubio F. (2019). Modulation of K^+^ translocation by AKT1 and AtHAK5 in Arabidopsis plants. Plant Cell Environ..

[32] Qi Z., Hampton C.R., Shin R., Barkla B.J., White P.J., Schachtman D.P. (2008). The high affinity K^+^ transporter AtHAK5 plays a physiological role in planta at very low K^+^ concentrations and provides a caesium uptake pathway in Arabidopsis. J. Exp. Bot..

[33] Pyo Y.J., Gierth M., Schroeder J.I., Cho M.H. (2010). High-affinity K^+^ transport in Arabidopsis: AtHAK5 and AKT1 are vital for seedling establishment and postgermination growth under low-potassium conditions. Plant Physiol..

[34] Sun Y.L., Kong X.P., Li C.L., Liu Y.X., Ding Z.J. (2015). Potassium retention under salt stress is associated with natural variation in salinity tolerance among Arabidopsis accessions. PLoS ONE.

[35] Okada T., Yamane S., Yamaguchi M., Kato K., Shinmyo A., Tsunemitsu Y., Iwasaki K., Ueno D., Demura T. (2018). Characterization of rice KT/HAK/KUP potassium transporters and K^+^ uptake by HAK1 from Oryza sativa. Plant Biotechnol..

[36] Chen G., Hu Q.D., Luo L., Yang T.Y., Zhang S., Hu Y.B., Yu L., Xu G.H. (2015). Rice potassium transporter OsHAK1 is essential for maintaining potassium-mediated growth and functions in salt tolerance over low and high potassium concentration ranges. Plant Cell Environ..

[37] Chen G., Zhang Y., Ruan B.P., Guo L.B., Zeng D.L., Gao Z.Y., Zhu L., Hu J., Ren D.Y., Yu L. (2018). OsHAK1 controls the vegetative growth and panicle fertility of rice by its effect on potassium-mediated sugar metabolism. Plant Sci..

[38] Yang T.Y., Zhang S., Hu Y.B., Wu F.C., Hu Q.D., Chen G., Cai J., Wu T., Moran N., Yu L. (2014). The role of a potassium transporter OsHAK5 in potassium acquisition and transport from roots to shoots in rice at low potassium supply levels. Plant Physiol..

[39] Horie T., Sugawara M., Okada T., Taira K., Kaothien-Nakayama P., Katsuhara M., Shinmyo A., Nakayama H. (2011). Rice sodium-insensitive potassium transporter, OsHAK5, confers increased salt tolerance in tobacco BY2 cells. J. Biosci. Bioeng..

[40] Bañuelos M.A., Garciadeblas B., Cubero B., Rodrıguez-Navarro A. (2002). Inventory and functional characterization of the HAK potassium transporters of rice. Plant Physiol..

[41] Wang X.H., Li J.F., Li F., Pan Y., Cai D., Mao D.D., Chen L.B., Luan S. (2021). Rice potassium transporter OsHAK8 mediates K^+^ uptake and translocation in response to low K^+^ stress. Front. Plant Sci..

[42] Zhang L.N., Sun X.Y., Li Y.F., Luo X., Song S.W., Chen Y., Wang X.H., Mao D.D., Chen L.B., Luan S. (2021). Rice Na^+^-permeable transporter OsHAK12 mediates shoots Na^+^ exclusion in response to salt stress. Front. Plant Sci..

[43] Feng H.M., Tang Q., Cai J., Xu B.C., Xu G.H., Yu L. (2019). Rice OsHAK16 functions in potassium uptake and translocation in shoot, maintaining potassium homeostasis and salt tolerance. Planta.

[44] Peng L.R., Xiao H.J., Li R., Zeng Y., Gu M., Moran N., Yu L., Xu G.H. (2023). Potassium transporter OsHAK18 mediates potassium and sodium circulation and sugar translocation in rice. Plant Physiol..

[45] Shen Y., Shen L.K., Shen Z.X., Jing W., Ge H.L., Zhao J.Z., Zhang W.H. (2015). The potassium transporter OsHAK21 functions in the maintenance of ion homeostasis and tolerance to salt stress in rice. Plant Cell Environ..

[46] Li W.H., Li M.Q., Li S., Zhang Y.F., Li X., Xu G.H., Yu L. (2022). Function of rice high-affinity potassium transporters in pollen development and fertility. Plant Cell Physiol..

[47] Almeida P., Katschnig D., de Boer A.H. (2013). HKT transporters--state of the art. Int. J. Mol. Sci..

[48] Wang R., Jing W., Xiao L.Y., Jin Y.K., Shen L.K., Zhang W.H. (2015). The rice high-affinity potassium transporter1;1 is involved in salt tolerance and regulated by an MYB-Type transcription factor. Plant Physiol..

[49] Garciadeblás B., Senn M.E., Bañuelos M.A., Navarro A.R. (2003). Sodium transport and HKT transporters: The rice model. Plant J..

[50] Suzuki K., Yamaji N., Costa A., Okuma E., Kobayashi N.I., Kashiwagi T., Katsuhara M., Wang C., Tanoi K., Murata Y. (2016). OsHKT1;4-mediated Na^+^ transport in stems contributes to Na^+^ exclusion from leaf blades of rice at the reproductive growth stage upon salt stress. BMC Plant Biol..

[51] Ren Z.H., Gao J.P., Li L.G., Cai X.L., Huang W., Chao D.Y., Zhu M.Z., Wang Z.Y., Luan S., Lin H.X. (2005). A rice quantitative trait locus for salt tolerance encodes a sodium transporter. Nat. Genet..

[52] Horie T., Costa A., Kim T.H., Han M.J., Horie R., Leung H.Y., Miyao A., Hirochika H., An G., Schroeder J.I. (2007). Rice OsHKT2;1 transporter mediates large Na^+^ influx component into K^+^-starved roots for growth. EMBO J..

[53] Lan W.Z., Wang W., Wang S.M., Li L.G., Buchanan B.B., Lin H.X., Gao J.P., Luan S. (2010). A rice high-affinity potassium transporter (HKT) conceals a calcium-permeable cation channel. Proc. Natl. Acad. Sci. USA.

[54] Yokoi S., Quintero F.J., Cubero B., Ruiz M.T., Bressan R.A., Hasegawa P.M., Pardo J.M. (2002). Differential expression and function of Arabidopsis thaliana NHX Na^+^/H^+^ antiporters in the salt stress response. Plant J..

[55] Fukuda A., Nakamura A., Hara N., Toki S., Tanaka Y. (2011). Molecular and functional analyses of rice NHX-type Na^+^ /H^+^ antiporter genes. Planta.

[56] Steudle E. (2001). The cohesion-tension mechanism and the acquisition of water by plant roots. Annu. Rev. Plant Physiol. Plant Mol. Biol..

[57] Hosmani P.S., Kamiya T., Danku J., Naseer S., Geldner N., Guerinot M.L., Salt D.E. (2013). Dirigent domain-containing protein is part of the machinery required for formation of the lignin-based Casparian strip in the root. Proc. Natl. Acad. Sci. USA.

[58] Kamiya T., Borghi M., Wang P., Danku J.M.C., Kalmbach L., Hosmani P.S., Naseer S., Fujiwara T., Geldner N., Salt D.E. (2015). The MYB36 transcription factor orchestrates Casparian strip formation. Proc. Natl. Acad. Sci. USA.

[59] Wu Q., Feng Z., Tsukagoshi H., Yang M., Ao Y., Fujiwara T., Kamiya T. (2023). Early differentiation of Casparian strip mediated by nitric oxide is required for efficient K transport under low K conditions in Arabidopsis. Plant J..

[60] Geldner N. (2013). The endodermis. Annu. Rev. Plant Biol..

[61] Tagliani A., Tran A.N., Novi G., Mambro R.D., Pesenti M., Sacchi G.A., Perata P., Pucciariello C. (2020). The calcineurin β-like interacting protein kinase CIPK25 regulates potassium homeostasis under low oxygen in Arabidopsis. J. Exp. Bot..

[62] Epstein E., Rains D.W., Elzam O.E. (1963). Resolution of dual mechanisms of potassium absorption by barley roots. Proc. Natl. Acad. Sci. USA.

[63] Gireth M., Maser P., Schroeder J.I. (2005). The potassium transpoters AtHAK5 functions in K^+^ deprivation-induced high-affinity K^+^ uptake and AKT1 K^+^ channel contribution to K^+^ uptake kinetics in Arabidopsis roots. Plant Physiol..

[64] Kochian L.V., Lucas W.J. (1982). Potassium transport in corn roots: I. resolution of kinetics into a saturable and linear component. Plant Physiol..

[65] Maathuis F.J.M. (2009). Physiological functions of mineral macronutrients. Curr. Opin. Plant Biol..

[66] Lagarde D., Basset M., Lepetit M., Conejero G., Gaymard F., Astruc S., Grignon C. (1996). Tissue-specific expression of Arabidopsis AKT1 gene is consistent with a role in K^+^ nutrition. Plant J..

[67] Ahmad I., Maathuis F.J.M. (2014). Cellular and tissue distribution of potassium: Physiological relevance, mechanisms and regulation. J. Plant Physiol..

[68] Liu K., Li L.G., Luan S. (2006). Intracellular K^+^ sensing of SKOR, a Shaker-type K^+^ channel from Arabidopsis. Plant J..

[69] Drechsler N., Zheng Y., Bohner A., Nobmann B., von Wirén N., Kunze R., Rausch C. (2015). Nitrate-dependent control of shoot K homeostasis by the nitrate transporter1/peptide transporter family member NPF7.3/NRT1.5 and the stelar K^+^ outward rectifier SKOR in Arabidopsis. Plant Physiol..

[70] Han M., Wu W., Wu W.H., Wang Y. (2016). Potassium transporter KUP7 is involved in K^+^ acquisition and translocation in Arabidopsis root under K^+^-limited conditions. Mol. Plant..

[71] Xia X.D., Fan X.R., Wei J., Feng H.M., Qu H.Y., Xie D., Miller A.J., Xu G.H. (2015). Rice nitrate transporter OsNPF2.4 functions in low-affinity acquisition and long-distance transport. J. Exp. Bot..

[72] Cuin T.A., Dreyer I., Michard E. (2018). The role of potassium channels in Arabidopsis thaliana long distance electrical signalling: AKT2 modulates tissue excitability while GORK shapes action potentials. Int. J. Mol. Sci..

[73] Held K., Pascaud F., Eckert C., Gajdanowicz P., Hashimoto K., Corratgé-Faillie C., Offenborn J.N., Lacombe B., Dreyer I., Thibaud J.B. (2011). Calcium-dependent modulation and plasma membrane targeting of the AKT2 potassium channel by the CBL4/CIPK6 calcium sensor/protein kinase complex. Cell Res..

[74] Kwak J.M., Murata Y., Baizabal-Aguirre V.M., Merrill J., Wang M., Kemper A., Hawke S.D., Tallman G., Schroeder J.I. (2001). Dominant negative guard cell K^+^ channel mutants reduce inward-rectifying K^+^ currents and light-induced stomatal opening in Arabidopsis. Plant Physiol..

[75] Pilot G., Lacombe B., Gaymard F., Chérel I., Boucherez J., Thibaud J.B., Sentenac H. (2001). Guard cell inward K^+^ channel activity in Arabidopsis involves expression of the twin channel subunits KAT1 and KAT2. J. Biol. Chem..

[76] Hosy E., Vavasseur A., Mouline K., Dreyer I., Gaymard F., Porée F., Boucherez J., Lebaudy A., Bouchez D., Véry A.A. (2003). The Arabidopsis outward K^+^ channel GORK is involved in regulation of stomatal movements and plant transpiration. Proc. Natl. Acad. Sci. USA.

[77] Wang R., Dobritsa A.A. (2018). Exine and aperture patterns on the pollen surface: Their formation and roles in plant reproduction. Annu. Plant Rev..

[78] Lu Y.X., Chanroj S., Zulkifli L., Johnson M.A., Uozumi N., Sez C.H. (2011). Pollen tubes lacking a pair of K^+^ transporters fail to target ovules in Arabidopsis. Plant Cell.

[79] Padmanaban S., Czerny D.D., Levin K.A., Leydon A.R., Su R.T., Maugel T.K., Zou Y., Chanroj S., Cheung A.Y., Johnson M.A. (2017). Transporters involved in pH and K^+^ homeostasis affect pollen wall formation, male fertility, and embryo development. J. Exp. Bot..

[80] Zhao L.N., Shen L.K., Zhang W.Z., Zhang W., Wang Y., Wu W.H. (2013). Ca_2_^+^-dependent protein kinase11 and 24 modulate the activity of the inward rectifying K^+^ channels in Arabidopsis pollen tubes. Plant Cell.

[81] Li D.D., Guan H., Li F., Liu C.Z., Dong Y.X., Zhang X.S., Gao X.Q. (2017). Arabidopsis shaker pollen inward K^+^ channel SPIK functions in SnRK1 complex-regulated pollen hydration on the stigma. J. Interg. Plant Biol..

[82] Amien S., Kliwer I., Marton M.L., Debener T., Geiger D., Becker D., Dresselhaus T. (2010). Defensin-like ZmES4 mediates pollen tube burst in maize via opening of the potassium channel KZM1. PLoS Biol..

[83] Yang F., Wang T., Liu L.T. (2020). Pollen germination is impaired by disruption of a Shaker K^+^ channel OsAKT1.2 in rice. J. Plant Physiol..

[84] Becker D., Geiger D., Dunkel M., Roller A., Bertl A., Latz A., Carpaneto A., Dietrich P., Roelfsema M.R.G., Voelker C. (2004). AtTPK4, an Arabidopsis tandem-pore K^+^ channel, poised to control the pollen membrane voltage in a pH-and Ca_2_^+^-dependent manner. Proc. Natl. Acad. Sci. USA.

[85] Haque U.S., Elias S.M., Jahan I., Seraj Z.I. (2023). Functional genomic analysis of K^+^ related salt-responsive transporters in tolerant and sensitive genotypes of rice. Front. Plant Sci..

[86] Liu L.T., Zheng C.H., Kuang B.J., Wei L.Q., Yan L.F., Wang T. (2016). Receptor-like kinase RUPO interacts with potassium transporters to regulate pollen tube growth and integrity in rice. PLoS Genet..

[87] Li L.G., Kim B.G., Cheong Y.H., Pandey G.K., Luan S. (2006). A Ca_2_^+^ signaling pathway regulates a K^+^ channel for low-K response in Arabidopsis. Proc. Natl. Acad. Sci. USA.

[88] Xu J., Li H.D., Chen L.Q., Wang Y., Liu L.L., He L., Wu W.H. (2006). A protein kinase, interacting with two calcineurin B-like proteins, regulates K^+^ transporter AKT1 in Arabidopsis. Cell.

[89] Tang R.J., Zhao F.G., Yang Y., Wang C., Li K.L., Kleist T.J., Lemaux P.G., Luan S. (2020). A calcium signalling network activates vacuolar K^+^ remobilization to enable plant adaptation to low-K environments. Nat. Plants.

[90] Ragel P., Ródenas R., García-Martín E., Andrés Z., Villalta I., Nieves-Cordones M., Rivero R.M., Martínez V., Pardo J.M., Quintero F.J. (2015). CIPK23 regulates HAK5-mediated high-affinity K^+^ uptake in Arabidopsis roots. Plant Physiol..

[91] Santa-María G.E., Oliferuk S., Moriconi J.I. (2018). KT-HAK-KUP transporters in major terrestrial photosynthetic organisms: A twenty years tale. J. Plant Physiol..

[92] Lhamo D., Wang C., Gao Q.F., Luan S. (2021). Recent advances in genome-wide analyses of plant potassium transporter families. Curr. Genom..

[93] Zhao S., Zhang M.L., Ma T.L., Wang Y. (2016). Phosphorylation of ARF2 relieves its repression of transcription of the K^+^ transporter gene HAK5 in response to low potassium stress. Plant Cell.

[94] Feng C.Z., Luo Y.X., Wang P.D., Gilliham M., Long Y. (2021). MYB77 regulates high-affinity potassium uptake by promoting expression of HAK5. New Phytol..

[95] Du X.Q., Wang F.L., Li H., Jing S., Yu M., Li J.G., Wu W.H., Kudla J., Wang Y. (2019). The transcription factor MYB59 regulates K^+^/${NO}_{3}^{-}$ translocation in the Arabidopsis response to low K^+^ stress. Plant Cell.

[96] Li G.Z., Liu J., Chen S.J., Wang P.F., Liu H.T., Dong J., Zheng Y.X., Xie Y.X., Wang C.Y., Guo T.C. (2021). Melatonin promotes potassium deficiency tolerance by regulating HAK1 transporter and its upstream transcription factor NAC71 in wheat. J. Pineal Res..

[97] Sheng H., Cong D.L., Ju H.Y. (2020). Functional characterization of ZmHAK1 promoter and its regulatory transcription factors in maize. Mol. Biol..

[98] Huang Y.N., Yang S.Y., Li J.L., Wang S.F., Wang J.J., Hao D.L., Su Y.H. (2021). The rectification control and physiological relevance of potassium channel OsAKT2. Plant Physiol..

[99] Song T.Z., Shi Y.Y., Shen L.K., Cao C.J., Shen Y., Jing W., Tian Q.X., Lin F., Li W.Y., Zhang W.H. (2021). An endoplasmic reticulum-localized cytochrome b5 regulates high-affinity K^+^ transport in response to salt stress in rice. Proc. Natl. Acad. Sci. USA.

[100] Riedelsberger J., Miller J.K., Valdebenito-Maturana B., Piñeros M.A., González W., Dreyer I. (2021). Plant HKT channels: An updated view on structure, function and gene regulation. Int. J. Mol. Sci..

[101] Wang J., Nan N., Li N., Liu Y.T., Wang T.J., Hwang I., Liu B., Xu Z.Y. (2020). A DNA methylation reader-chaperone regulator-transcription factor complex activates OsHKT1;5 expression during salinity stress. Plant Cell.

[102] Xiao L.Y., Shi Y.Y., Wang R., Feng Y., Wang L.S., Zhang H.S., Shi X.Y., Jing G.Q., Deng P., Song T.Z. (2022). The factor OsMYBc and an E3 ligase regulate expression of a K^+^ transporter during salt stress. Plant Physiol..

[103] Shabala S., Cuin T.A. (2008). Potassium transport and plant salt tolerance. Physiol. Plant..

[104] Munns R., Tester M. (2008). Mechanisms of salinity tolerance. Annu. Rev. Plant Biol..

[105] Anschutz U., Becker D., Shabala S. (2014). Going beyond nutrition: Regulation of potassium homoeostasis as a common denominator of plant adaptive responses to environment. J. Plant Physiol..

[106] Ismail A.M., Horie T. (2017). Genomics, physiology, and molecular breeding approaches for improving salt tolerance. Annu. Rev. Plant Biol..

[107] Zhang M., Liang X.Y., Wang L.M., Cao Y.B., Song W.B., Shi J.P., Lai J.S., Jiang C.F. (2019). A HAK family Na^+^ transporter confers natural variation of salt tolerance in maize. Nat. Plants.

[108] Cuin T.A., Bose J., Stefano G., Jha D., Tester D., Mancuso S., Shabala S. (2011). Assessing the role of root plasma membrane and tonoplast Na^+^/H^+^ exchangers in salinity tolerance in wheat: In planta quantification methods. Plant Cell Environ..

[109] Roy S.J., Negrão S., Tester M. (2014). Salt resistant crop plants. Curr. Opin. Biotechnol..

[110] Zhang Y.Y., Wang L.L., Liu Y.L., Zhang Q., Wei Q.P., Zhang W.H. (2006). Nitric oxide enhances salt tolerance in maize seedlings through increasing activities of proton-pump and Na^+^/H^+^ antiport in the tonoplast. Planta.

[111] Mian A., Oomen R.J.F.J., Isayenkov S., Sentenac I.H., Maathuis F.J.M., Véry A.A. (2011). Over-expression of an Na^+^- and K^+^-permeable HKT transporter in barley improves salt tolerance. Plant J..

[112] Gong J.M., Waner D.A., Horie T., Li S.L., Horie R., Abid K.B., Schroeder J.I. (2004). Microarray-based rapid cloning of an ion accumulation deletion mutant in Arabidopsis thaliana. Proc. Natl. Acad. Sci. USA.

[113] Wei H., Wang X.L., He Y.Q., Xu H., Wang L. (2021). Clock component OsPRR73 positively regulates rice salt tolerance by modulating OsHKT2;1-mediated sodium homeostasis. EMBO J..

[114] Davenport R.J., Muñoz-Mayor A., Jha D., Essah P.A., Rus A., Tester M. (2007). The Na^+^ transporter AtHKT1;1 controls retrieval of Na^+^ from the xylem in Arabidopsis. Plant Cell Environ..

[115] Sunarpi X., Horie T., Motoda J., Kubo M., Yang H., Yoda K., Horie R., Chan W.Y., Leung H.Y., Hattori K. (2005). Enhanced salt tolerance mediated by AtHKT1 transporter-induced Na unloading from xylem vessels to xylem parenchyma cells. Plant J..

[116] Pruthi R., Chapagain S., Coronejo S., Singh L., Subudhi P.K. (2022). Quantitative trait loci, candidate genes, and breeding lines to improve salt tolerance at the flowering and seedling stages in rice. Food Energy Secur..

[117] Obata T., Kitamoto H.K., Nakamura A., Fukuda A., Tanaka Y. (2007). Rice shaker potassium channel OsKAT1 confers tolerance to salinity stress on yeast and rice cells. Plant Physiol..

[118] Leidi E.O., Barragán V., Rubio L., El-Hamdaoui A., Ruiz M.T., Cubero B., Fernández J.A., Bressan R.A., Hasegawa P.M., Quintero F.J. (2010). The AtNHX1 exchanger mediates potassium compartmentation in vacuoles of transgenic tomato. Plant J..

[119] Barragán V., Leidi E.O., Andrés Z., Rubio L., Luca A.D., Fernández J.A., Cubero B., Pardo J.M. (2012). Ion exchangers NHX1 and NHX2 mediate active potassium uptake into vacuoles to regulate cell turgor and stomatal function in Arabidopsis. Plant Cell.

[120] Bassil E., Ohto M., Esumi T., Tajima H., Zhu Z., Cagnac O., Belmonte M., Peleg Z., Yamaguchi T., Blumwald E. (2011). The Arabidopsis intracellular Na^+^/H^+^ antiporters NHX5 and NHX6 are endosome associated and necessary for plant growth and development. Plant Cell.

[121] Zelm E.V., Zhang Y.X., Testerink C. (2020). Salt tolerance mechanisms of plants. Annu. Rev. Plant Biol..

[122] Halfter U., Ishitani M., Zhu J.K. (2000). The Arabidopsis SOS2 protein kinase physically interacts with and is activated by the calcium-binding protein SOS3. Proc. Natl. Acad. Sci. USA.

[123] Lu K.K., Song R.F., Guo J.X., Zhang Y., Zuo J.X., Chen H.H., Liao C.Y., Hu X.Y., Ren F., Lu Y.T. (2023). CycC1;1-WRKY75 complex-mediated transcriptional regulation of SOS1 controls salt stress tolerance in Arabidopsis. Plant Cell.

[124] Zhang X.Y., Tang L.H., Nie J.W., Zhang C.R., Han X.N., Li Q.Y., Qin L., Wang M.H., Huang X.H., Yu F.F. (2023). Structure and activation mechanism of the rice Salt Overly Sensitive 1 (SOS1) Na^+^/H^+^ antiporter. Nat. Plants.

[125] Zhang Q.J., Li G.H., Lu W.P., Lu D.L. (2022). Interactive effects of nitrogen and potassium on grain yield and quality of waxy maize. Plants.

[126] Meng S., Peng J.S., He Y.N., Zhang G.B., Yi H.Y., Fu Y.L., Gong J.M. (2016). Arabidopsis NRT1.5 mediates the suppression of nitrate starvation-induced leaf senescence by modulating foliar potassium level. Mol. Plant..

[127] Straub T., Ludewig U., Neuhäuser B. (2017). The Kinase CIPK23 inhibits ammonium transport in Arabidopsis thaliana. Plant Cell.

[128] Bao A.L., Liang Z.J., Zhao Z.Q., Cai H.M. (2015). Overexpressing of OsAMT1-3, a high affinity ammonium transporter gene, modifies rice growth and carbon-nitrogen metabolic status. Int. J. Mol. Sci..

[129] Wei J., Zheng Y., Feng H.M., Qu H.Y., Fan X.R., Yamaji N., Ma J.F., Xu G.H. (2018). OsNRT2.4 encodes a dual-affinity nitrate transporter and functions in nitrate-regulated root growth and nitrate distribution in rice. J. Exp. Bot..

[130] Dai S.H., Wu H.C., Chen H.Y., Wang Z.H., Yu X., Wang L., Jia X.Q., Qin C., Zhu Y.Y., Yi K.K. (2023). Comparative transcriptome analyses under individual and combined nutrient starvations provide insights into N/P/K interactions in rice. Plant Physiol. Bioch..

[131] Wang Y.H., Garvin D.F., Kochian L.V. (2002). Rapid induction of regulatory and transporter genes in response to phosphorus, potassium, and iron deficiencies in tomato roots. Evidence for cross talk and root/rhizosphere-mediated signals. Plant Physiol..

[132] Zhang F., Sun Y.F., Pei W.X., Jain A., Sun R., Cao Y., Wu X.N., Jiang T.T., Zhang L., Fan X.R. (2015). Involvement of OsPht1;4 in phosphate acquisition and mobilization facilitates embryo development in rice. Plant J..

